# Requirement of LaeA, VeA, and VelB on Asexual Development, Ochratoxin A Biosynthesis, and Fungal Virulence in *Aspergillus ochraceus*

**DOI:** 10.3389/fmicb.2019.02759

**Published:** 2019-11-28

**Authors:** Gang Wang, Haiyong Zhang, Yulong Wang, Fei Liu, Erfeng Li, Junning Ma, Bolei Yang, Chenxi Zhang, Li Li, Yang Liu

**Affiliations:** ^1^Key Laboratory of Agro-products Quality and Safety Control in Storage and Transport Process, Ministry of Agriculture and Rural Affairs, Institute of Food Science and Technology, Chinese Academy of Agricultural Sciences, Beijing, China; ^2^Horticulture and Landscape College, Tianjin Agricultural University, Tianjin, China

**Keywords:** *Aspergillus ochraceus*, ochratoxin A, LaeA, VeA, VelB, secondary metabolism, development, virulence

## Abstract

*Aspergillus ochraceus* is reported to be the major contributor of ochratoxin A (OTA), classified as one of the possible human carcinogen (group 2B) by the International Agency for Research on Cancer. The heterotrimeric velvet complex proteins, LaeA/VeA/VelB, have been most studied in fungi to clarify the relation between light-dependent morphology and secondary metabolism. To explore possible genetic targets to control OTA contamination, we have identified laeA, veA, and velB in *A. ochraceus*. The loss of *laeA*, *veA*, and *velB* yielded mutants with differences in vegetative growth and conidial production. Especially, ΔlaeA almost lost the ability to generate conidiaphore under dark condition. The deletion of *laeA*, *veA*, and *velB* drastically reduced the production of OTA. The wild-type *A. ochraceus* produced about 1 and 7 μg/cm^2^ OTA under light and dark conditions on media, whereas the three gene deletion mutants produced less than 20 ng/cm^2^ OTA, which was correlated with a down regulation of OTA biosynthetic genes. Pathogenicity studies of ΔlaeA, ΔveA, and ΔvelB showed their reduction in disease severity in pears. Furthermore, 66.1% of the backbone genes in secondary metabolite gene cluster were significantly regulated, among which 81.6% were downregulated. Taking together, these results revealed that velvet complex proteins played crucial roles in asexual development, secondary metabolism, and fungal virulence in *A. ochraceus*.

## Introduction

Ochratoxin A (OTA) is the secondary metabolite of *Aspergillus* and *Penicillium* species (Wang et al., 2016a,b). That poses a serious health hazard according to its mycotoxic properties (Taniwaki et al., 2018). It is classified as a possible human carcinogen (group 2B) by the International Agency for Research on Cancer (IARC, 1993). OTA was first isolated from *A. ochraceus* in 1965 (van der Merwe et al., 1965). And it was reported to be the major contributor of OTA in cereal, *Zea mays*, coffee, fruits, and beverage (Mantle, 2002).

The biosynthetic pathway of OTA has been extensively studied in the past decades (William and Hamilton, 1979; Wang et al., 2015; Gallo et al., 2017; Geisen et al., 2018). Wang has identified a conserved OTA biosynthetic gene cluster by comparatively analysis of six OTA-producing fungi and clarified its biosynthetic pathway by deletion mutants of four structural genes (*otaA*, *B*, *C*, and *D*) and one regulatory gene (*otaR1*) (Wang et al., 2018a,b). Environmental factors are crucial to regulation of OTA production (Selouane et al., 2009; Abarca et al., 2019). The mechanism of OTA biosynthesis is very complex and acts at different levels. Generally, environmental signals transmit to biosynthetic cluster to activate/repress the production of OTA by global regulators and multiprotein complexes. For example, *Aoyap1*, a transcription factor related to oxidative stress, regulated OTA synthesis by controlling cell redox balance in *A. ochraceus* (Reverberi et al., 2012). The transcriptional factors *AopacC* (Wang et al., 2018a,b) and *hog* (Schmidt-Heydt et al., 2012) that are functionally performed pH signaling and osmotic stress were also involved in the regulatory mechanism of OTA biosynthesis at pH stress and osmotic stress, respectively. The heterotrimeric velvet complex, VelB/VeA/LaeA, has been most studied in fungi to clarify the relation between light-dependent morphology and secondary metabolism. In *A. nidulans*, VeA bridges VelB to LaeA, the nuclear master regulator of secondary metabolism (Bayram et al., 2008). LaeA has also been suggested as an epigenetic regulator for its methyltransferase functions toward amino acid lysine and arginine. Several structure homologous LaeA proteins have been identified in *A. fumigatus* (Bok et al., 2005), *A. oryzae* (Oda et al., 2011), *Cochliobolus heterostrophus* (Wu et al., 2012), *Fusarium oxysporum* (Lopez-Berges et al., 2014), *P. chrysogenum* (Veiga et al., 2012), and *Trichoderma reesei* (Karimi-Aghcheh et al., 2013) and demonstrated profound influence on sporulation capacity, mycelial growth, sclerotia formation, and secondary metabolite production.

Several studies have been conducted to regulate mycotoxin biosynthesis by LaeA. The deletion of *laeA* in *A. flavus* led to the loss of aflatoxin mediated by the expression loss of *aflR*, specific transcription factor in aflatoxin biosynthetic cluster. The conidial production, sclerotia formation, and host colonization were repressed in the ΔlaeA of *A. flavus* (Kale et al., 2008). Deletion of *laeA* and *veA* greatly reduced sporulation and strongly copromised the alternariol and alternariol monomethyl ether production (Estiarte et al., 2016). In *A. carbonarius*, Crespo-Sempere suggested that VeA and LaeA have an important role regulating conidiation and OTA biosynthesis (Crespo-Sempere et al., 2013). The *veA* gene was proven to act as a positive regulator of conidia production, OTA biosynthesis, and oxidative stress tolerance in *A. niger* (Zhang et al., 2018). *A. steynii*, *A. niger*, *P. nordicum*, and *P. verrucosum* were described about their ability to produce OTA response to light (Schmidt-Heydt et al., 2010, 2011). However, comprehensive study about velvet complex regulated OTA biosynthesis responding to light is needed.

There is still limited information regarding to the link of light and OTA biosynthesis and their regulatory mechanism in *A. ochraceus*, except Aziz reported white and UV light affected mycelial growth and OTA production in 1997 (Aziz and Moussa, 1997). Nothing has been reported about the function of velvet complex proteins in *A. ochraceus*. For this purpose, we have identified and deleted the members of velvet complex (laeA, veA, and velB) in *A. ochraceus* and explored their regulatory role in growth morphology, OTA biosynthesis and fungal virulence on pears. Furthermore, we demonstrated how LaeA affects secondary metabolism in *A. ochraceus* at gene expression level.

## Materials and Methods

### Strains and Growth Conditions

The wild type (WT) strain *A. ochraceus* fc-1 used in this study was isolated, characterized, and genome sequenced in our laboratory (Wang et al., 2018a,b). WT and mutant strains were routinely cultured at 28°C under dark condition. For phenotype and gene expression studies, all utilized strains were cultured on potato dextrose agar (PDA, BD DifcoTM, USA) at 28°C. Each strain was cultured on four plates as technical replicates, and each experiment was repeated three times as biological replicates.

### Phylogenetic Tree and Functional Analysis

LaeA, VeA, and VelB amino acid sequences from *A. nidulans* (Bayram et al., 2008), *A. flavus* (Kale et al., 2008) and *Cochliobolus heterostrophus* (Wu et al., 2012) were used as queries, and basic local alignment search tool algorithm was used to search LaeA, VeA, and VelB from the genome of *A. ochraceus*, *A. niger*, *A. welwitschiae*, *A. lacticoffeatus*, *A. sclerotioniger*, *A. steynii*, and *P. nordicum* from the National Center for Biotechnology Information resources (NCBI). The amino acid sequences of LaeA were aligned by MUSCLE, and a maximum likelihood phylogeny was constructed by treeBeST using 1,000 bootstrap replicates.

### Generation of Gene Deletion Mutants

To construct *laeA*, *veA*, and *velB* mutants, previous approach reported in our group was used, and the deletion cassettes were generated by overlap PCR procedures (Wang et al., 2018a,b). Primers utilized in this study were listed in Supplementary Table S1. And then fusion PCR products were transformed into the protoplasts of *A. ochraceus*. Transformants were verified by Southern blotting. Briefly, approximately 20 μg genomic DNA of each sample was complete-digested and separated 1% agarose gel and transferred to a Hybond-N+ nylon membrane (GE healthcare, UK). After alkali denaturation and neutralization, hybridization was detected with digoxigenin-labeled probes using DIG high-prime DNA labeling and detection starter Kit II (Roche, Basel, Switzerland) according to the instructions of the manufacturer. Primers for probe amplification were listed in Supplementary Table S1.

### Phenotypic Studies of Mutants

For mutant’s growth assessment, PDA plates were inoculated at center with 1 μl of conidia suspension (10^6^ conidia/ml) of each strain and cultures were incubated at 28°C for 9 days under two conditions, white light (Mazda, 23 W CFT/827, 1,485 lm) and darkness. The growth rate was analyzed by measuring the colony diameter of each mutant. For phenotypic study, the hyphae and spores were observed under optical microscope and electron microscope. For further analysis, conidia were collected from six agar plugs (1 cm diameter) from equivalent zones of fungal surface of PDA. The collected samples were homogenized and diluted in 0.1% Tween-80 and counted by a hemocytometer.

### Analysis of Ochratoxin A Production

For the investigation of OTA, WT, ΔlaeA, ΔveA, and ΔvelB of *A. ochraceus* were cultured on PDA for 9 days under light and dark conditions. Six agar plugs (1 cm diameter) from equivalent zones of fungal surface of PDA were collected and extracted with 6 ml methanol ultrasonically. Then, the supernatant was filtered through a 0.22 μm filter into a vial. Next, HPLC analysis was performed on an Agilent HPLC system for analyzing the concentration of OTA as previously described method (Wang et al., 2018a,b).

### Pathogenicity Assay

Fresh pears (*Pyrus × bretschneideri*) were selected to test the pathogenicity of WT and mutant strains of *A. ochraceus in vitro*. The upper surface of pears were disinfected three times with 0.1% sodium hypochlorite (NaClO) for 10 s and rinsed with sterilized water for 30 s. Each pear was punctured by sterilized needle to approximately 2 mm depth to make a wound (2 mm diameter) for inoculation, injected 2 μl conidia suspension (10^6^ conidia/ml) in wound, in contrast sterilized water was served as control and incubated at 28°C under dark condition. The diameter of scab was measured after 5 and 9 days.

### DNA and RNA Isolation

The mycelium of *A. ochraceus* strains were harvested *via* filtration. Genomic DNA was isolated using a Qiagen DNeasy kit, according to the manufacturer’s protocol. For RNA isolation, the *A. ochraceus* mycelium tissues were grown on PDA medium at 28°C for 9 days under light condition. RNA was extracted using TRIZOL reagent (Invitrogen, USA) following the manufacturer’s protocol.

### Real-Time Polymerase Chain Reaction Analysis and Quantitative Real-Time Polymerase Chain Reaction Analysis

Three biological replicates were performed for each analysis of the relative expression levels. Reverse transcription of 500 ng RNA was performed with a TIANScript II RT Kit (TIANGEN, China). The *A. ochraceus gadph* gene served as an internal standard. Primers for the RT-PCR amplification were listed in Supplementary Table S2. The cDNA was analyzed by qRT-PCR using SYBR Premix Ex Taq^™^ II (TAKARA) on a BIO-RAD CFX96 (BIO-RAD). The *gadph* gene serving as house-keeping gene was used for normalization. The relative expression values were calculated and the expression ratios were quantified using the 2^−∆∆Ct^ method. Primers were listed in Supplementary Table S3.

### Statistical Analysis

All data were analyzed with IBM SPSS statistics version 20 and presented with the means and standard deviation. The statistical significances among sample groups were calculated with ANOVA and means were compared by least significant difference (LSD) and Duncan’s test. The difference was regard to be statistic significant at *p* < 0.05.

## Results

### Identification, Analysis, and Disruption of LaeA, VeA, and VelB in *A. ochraceus*

**Figure 1:**
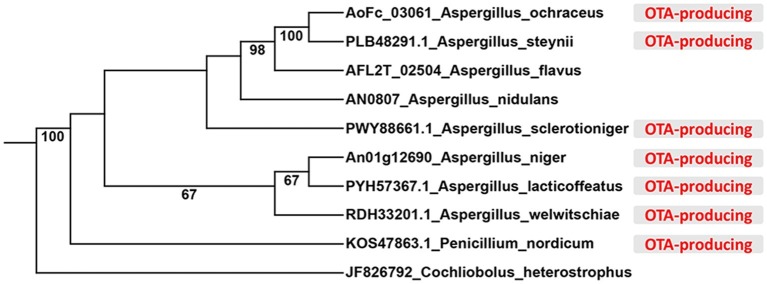
Phylogenetic relationship of LaeA protein from different species. The OTA-producing fungi were marked in red color.

**Figure 2:**
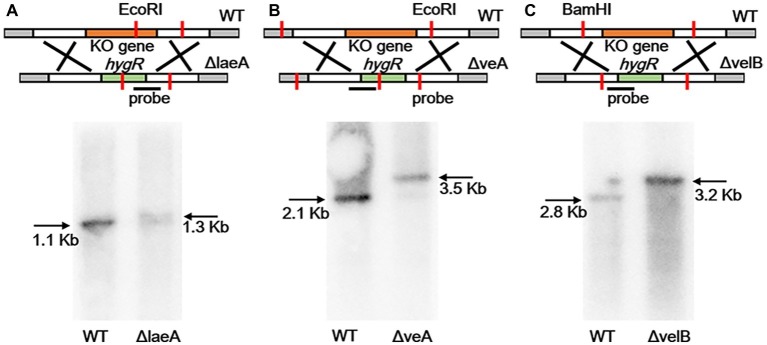
Southern blotting verification of laeA, veA, and velB gene deletion. **(A)** The WT and ΔlaeA isolates were digested with *EcoR*I. A fragment amplified from ΔlaeA was used as the probe. **(B)** The WT and ΔveA isolates were digested with *EcoR*I. A fragment amplified from ΔveA was used as the probe. **(C)** The WT and ΔvelB isolates were digested with *BamH*I. A fragment amplified from ΔvelB was used as the probe.

In order to identify velvet protein homologs in *A. ochraceus*, the genome sequence of *A. ochraceus* was interrogated using Blast alignment approach. BlastP searches were performed using LaeA, VeA, and VelB amino acid sequences from *A. nidulans*, *A. flavus* and *Cochliobolus heterostrophus* as the probes and the homologs AoFC_03061, AoFC_07220 and AoFC_09406 were identified. LaeA from *A. steynii* (XP_024703593.1), VeA from *A. tanneri* (THC96327.1), and VelB from *A. tanneri* (THC97134.1) were found to be most related to velvet complex proteins in *A. ochraceus*, with the identity of 95.2, 72.5 and 89.6%, respectively. A phylogenetic tree of evolutionary relationship of LaeA proteins from various species including OTA producing fungi was constructed (Figure 1), revealing that LaeA was conserved among the *Aspergillus* species. Inactivation of LaeA, VeA, and VelB locus was obtained by homologous replacement of the genes by encoding gene of hygromycin B phosphotransferase (*hygR*). The strategy of mutant generation was shown in Supplementary Figure S1A. The isolate resistant to hygromycin B was screened by PCR using primers in marker gene namely *hygR* and outside the knockout cassette (Supplementary Figure S1B). At least three transformants of each gene disrupted mutant were obtained from the mutant generation. Southern blot analysis also showed that *ΔlaeA* (Figure 2A), *ΔveA* (Figure 2B), and *ΔvelB* (Figure 2C) lack the target genes (*laeA, veA*, and *velB*).

### Involvement of LaeA, VeA, and VelB in Asexual Development, Growth Rate, and Conidiation

**Figure 3:**
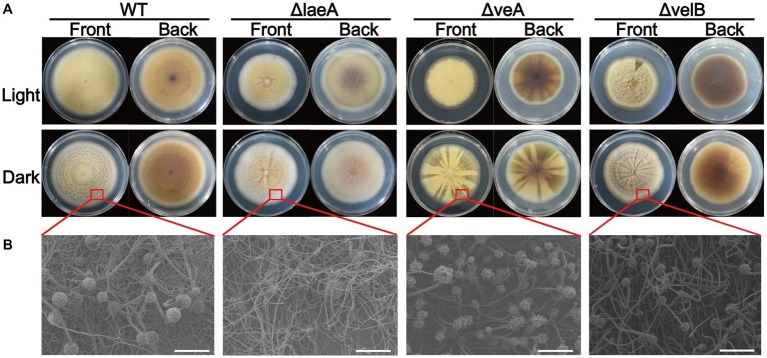
Colony view of the WT, ΔlaeA, ΔveA, and ΔvelB strains of *A. ochraceus*. **(A)** The front and back of *A. ochraceus* colony under light and dark conditions. **(B)** Scanning electron micrograph of *A. ochraceus* strains (scale bar = 200 μm). The red box represented the part of colony for observation.

A series of difference related to colony morphology, asexual development and conidiation were observed in ΔlaeA, ΔveA, and ΔvelB compared with the WT of *A. ochraceus* on PDA media under light and dark conditions. Under light condition as shown in Figure 3A, the WT colonies grew in yellow uniform layer while the laeA deletion mutant grew as a white-yellow cover. We also observed a pigment reduction for ΔlaeA, and a pigment increasing for ΔveA and ΔvelB in the back of the Petri dishes. Under dark condition, the WT *A. ochraceus* showed more pigmentation compared to the light condition. The ΔlaeA grew as a white color for the decrease of spores and pigment (Figure 3A). A reduction of conidiophore in ΔlaeA compared with the other strains from the colony edge under dark condition by scanning electron micrograph was observed (Figure 3B).

**Figure 4:**
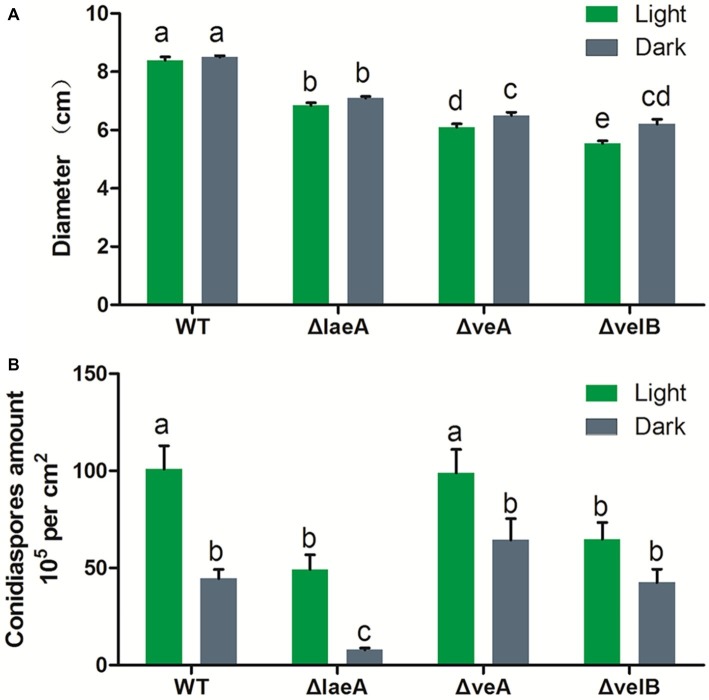
Effect of LaeA, VeA, and VelB deletion on the colony growth and conidiation of *A. ochraceus*. **(A)** Diameter of WT, ΔlaeA, ΔveA, and ΔvelB under light and dark conditions. **(B)** Conidiaspore production of WT, ΔlaeA, ΔveA, and ΔvelB under light and dark conditions. Different letters indicate a significant difference between the corresponding values (*p* < 0.05) with three biological replicates.

Light condition had no effect on the growth rate of *A. ochraceus* strains for WT and ΔlaeA, while repressing the growth of ΔveA and ΔvelB (*p* < 0.05). The growth rate was significantly decreased in ΔlaeA, ΔveA and ΔvelB compared with the WT (Figure 4A). Mycotoxin-producing fungi caused extensive infestations by generating asexual spores called conidiaspore. To investigate the involvement of LaeA, VeA and VelB in conidiation, the conidiaspore number was counted for strains cultured for 9 days under light and dark conditions. We found conidial generation was increased in the light condition for the *A. ochraceus* strains, although the conidiaspore amount of ΔvelB under light and dark condition demonstrated non-significant difference at statistic level (Figure 4B). The deletion of laeA resulted in a drastic reduction of conidial generation, whose inactivation leading to *A. ochraceus* almost loss the ability to generate conidiaspore under dark condition (Figure 4B). The conidiaphore amount of ΔlaeA and ΔvelB under light condition demonstrated significant difference compared with the WT. These results indicated the velvet complex proteins (LaeA, VeA and VelB) play important roles in colony phenotype, growth rate and conidiation.

### Requirement of LaeA, VeA, and VelB in Ochratoxin A Biosynthesis

**Figure 5:**
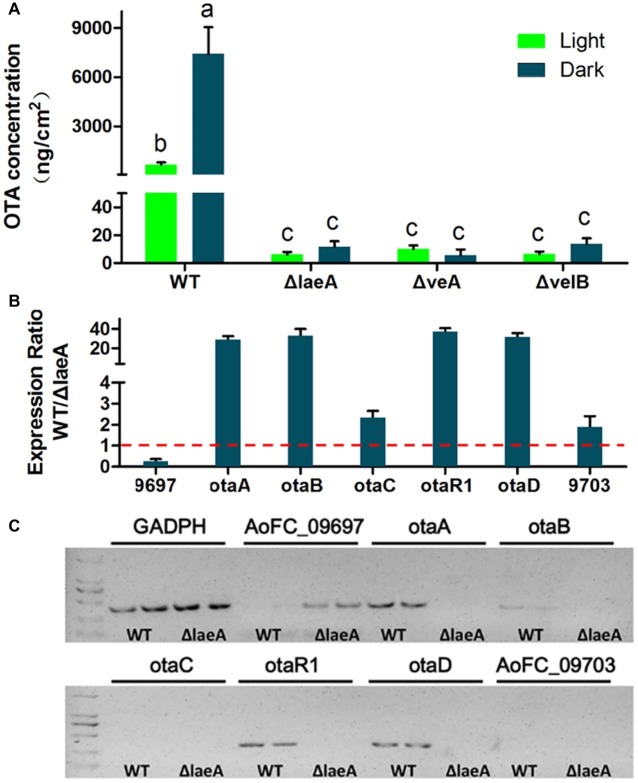
OTA production in WT, ΔlaeA, ΔveA, and ΔvelB of *A. ochraceus*. **(A)** OTA concentration in WT, ΔlaeA, ΔveA, and ΔvelB under light and dark conditions. Different letters indicate a significant difference between the corresponding values (*p* < 0.05) with three biological replicates. **(B)** qRT-PCR was run to check the expression ratio of the genes which are involved OTA biosynthesis and as well as present inside and outside of OTA biosynthetic gene cluster in WT and compared to ΔlaeA mutant. **(C)** RT-PCR amplification of the genes in and out OTA biosynthetic gene cluster.

In order to investigate whether LaeA, VeA, or VelB is linked to secondary metabolism related to OTA biosynthesis, the crude extractions of *A. ochraceus* of 9-day-old cultures were analyzed by HPLC. The results showed the deletion of *laeA*, *veA*, and *velB* drastically reduced the production of OTA. The WT *A. ochraceus* produced about 1and 7 μg/cm^2^ OTA under light and dark condition on media, while the three gene deletion mutants produced less than 20 ng/cm^2^ OTA (Figure 5A). We observed white light was an inhibitory factor for OTA biosynthesis. To further elucidate the function of LaeA as regulator of OTA biosynthesis, the expression level of genes in the OTA biosynthetic cluster was comparatively examined in WT and ΔlaeA in the dark condition. As shown in Figure 5B, the results of qRT-PCR analysis confirmed the expression level of *otaA*, *otaB*, *otaC*, *otaR1*, and *otaD* was downregulated 2–40-fold in ΔlaeA compared to those genes in WT. The upstream gene AoFC_09697 and downstream gene AoFC_09703 showed different expression profiles in WT and also *ΔlaeA* with respect to the OTA biosynthetic gene. The transcripts of the four OTA biosynthetic genes (*otaA*, *otaB*, *otaD*, and *otaR1*) were detected in WT by amplification by RT-PCR but not in ΔlaeA. The otaC gene was not detected in WT because of its low level of expression (Figure 5C). These results were consistent with the production of OTA, which could be detected in WT and could not be detected in ΔlaeA.

### Roles of LaeA, VeA, and VelB in Fungal Virulence

**Figure 6:**
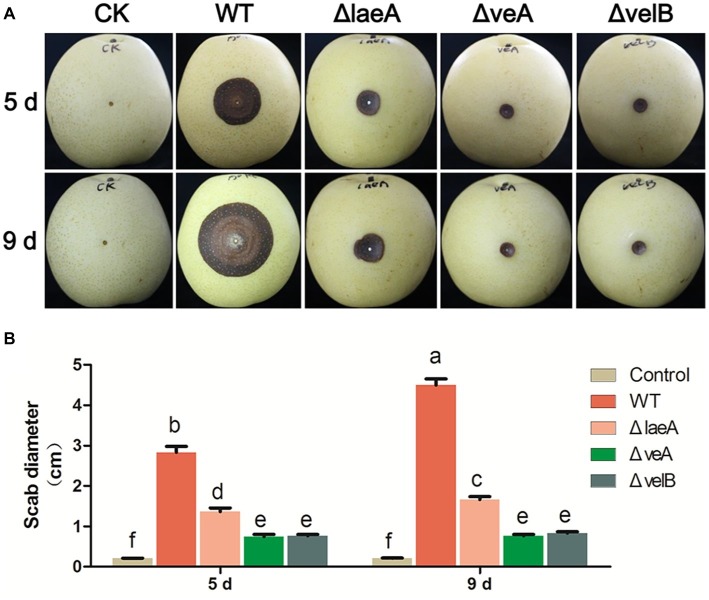
Pathogenicity assay for WT and mutants of *A. ochraceus* on pears. **(A)** Pears infected by WT, ΔlaeA, ΔveA, and ΔvelB incubated at 28°C for 5 and 9 days under dark condition and photographed. **(B)** The scab diameters of pears measured using cross method. Different letters indicate a significant difference between the corresponding values (*p* < 0.05) with three biological replicates.

The influence of LaeA, VeA, and VelB on the capacity of *A. ochraceus* to infect pears was ascertained. Lesion diameters were measured at 5 and 9 days after infection. After incubation for 5 days, lesions infected by all *A. ochraceus* strains were observed. Obviously, the lesions infected by ΔlaeA, ΔveA, and ΔvelB were repressed when compared with the lesions infected by WT (Figure 6A). Figure 6B demonstrated the significant difference in statistic level. After incubation for 9 days, the lesion infected by WT obviously increased. Lesions infected by ΔveA and ΔvelB had little change compared with incubation for 5 days. This study illustrated that the loss of velvet proteins would weaken the infection ability of *A. ochraceus* on pear.

### LaeA Extensively Regulated Secondary Metabolism in *A. ochraceus*

**Figure 7:**
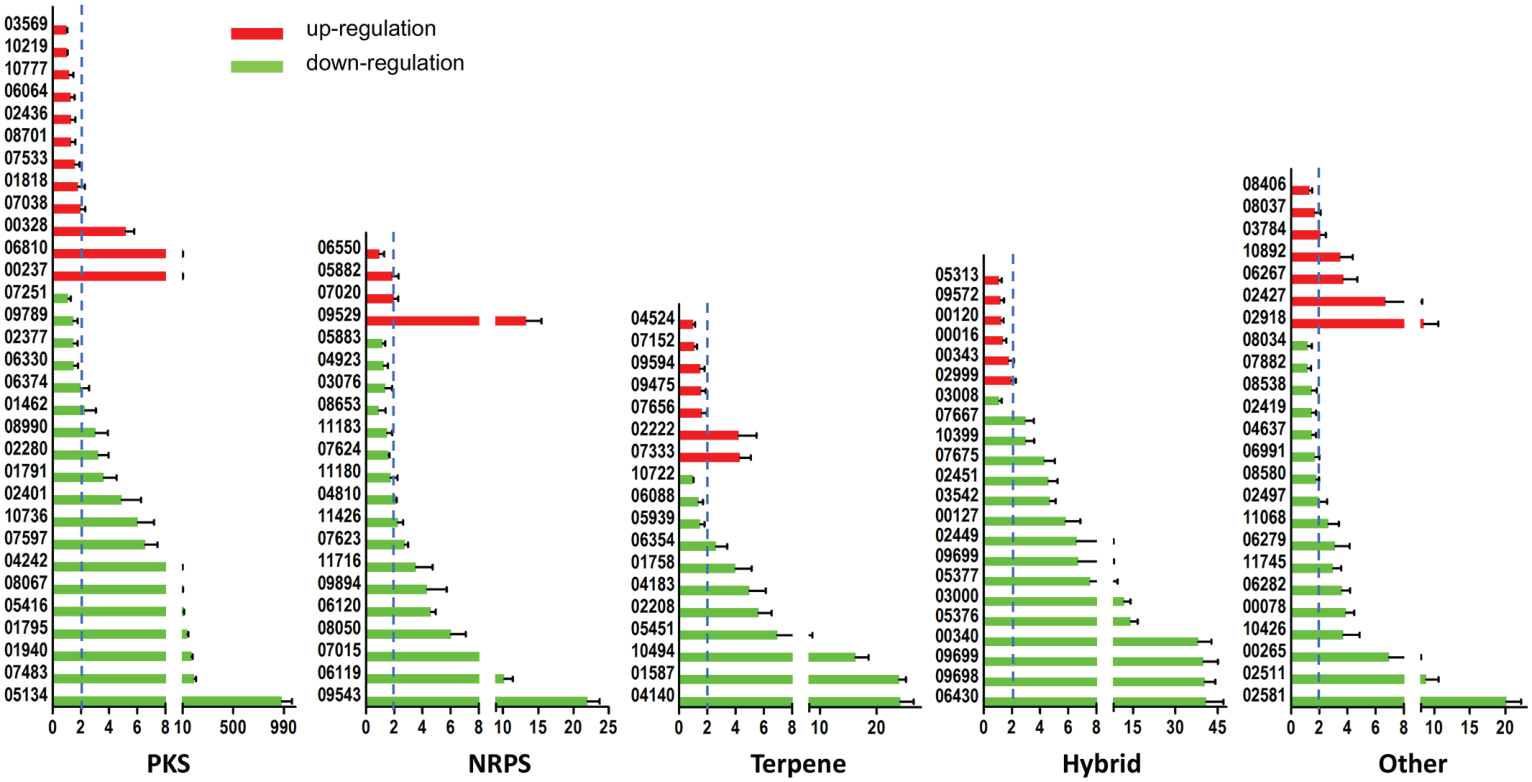
LaeA influenced the expression level of secondary metabolite biosynthetic genes. Both WT and ΔlaeA had three biological replicates. Y axes represented the backbone genes in PKS, NRPS, Terpene, Hybrid, and other gene clusters. X axes represented the expression ratio of genes expressed in WT compared to that expressed in ΔlaeA.

As earlier reported, the *A. ochraceus* genome contains 99 secondary metabolite biosynthetic gene clusters (Wang et al., 2018a,b). The expression level of backbone genes in secondary metabolites cluster were checked by qRT-PCR (Figure 7). About 66.1% of the backbone genes in the cluster were differentially expressed at *p* < 0.01, and 81.6% of the differential expression genes were down-regulated in *laeA* deletion mutant. About 58.6% of the backbone genes’ expression level were regulated at least two folds, among which 81.2% were down-regulated. These results indicated that LaeA was essential for the expression of considerable part of secondary metabolite encoding genes.

## Discussion

OTA contamination of food, feed, and fruits is a significant health concern worldwide. *A. ochraceus* is the major producer of OTA, with a wide range of host. Furthermore, a number of secondary metabolites, such as circumdatin G and H (Dai et al., 2001; Lopez-Gresa et al., 2005), stephacidin A and B (Jingfang Qian-Cutrone et al., 2002), Speramides A and B (Chang et al., 2016), and waspergillamide B (Frank et al., 2019), could be produced by *A. ochraceus* and researchers never give up to isolate new compounds from this fungus. However, the role of secondary metabolites except ochratoxins on health and virulence is unknown. And little is known about the genetic regulation of the lots of secondary metabolites including OTA biosynthesis process. Thus, deep inspection of the regulatory genes involved in metabolic pathways could provide a better understanding the mechanism of regulation of secondary metabolites.

In 2008, it was revealed that LaeA and two velvet families, VeA and VelB, confirmed a trimetirc complex that is essential to coordinate secondary metabolism and development in *A. nidulans* under dark condition (Bayram et al., 2008). VeA forms the light-responsive bridge that links VelB and LaeA. Three proteins were conserved in various fungi. In the WT of *A. ochraceus*, light cause a 50% increase of conidiospore and a 92% reduction of OTA. It is found that OTA biosynthesis was reduced under light condition for other ochratoxingenic fungi such as *A. carbonarius*, *A. niger*, *P. verrucosum*, and *P. nordicum* (Schmidt-Heydt et al., 2010; Crespo-Sempere et al., 2013), indicated that the development and secondary metabolism was regulated by light condition and might be explained according to the role of velvet complex. Here, we are reporting first time the function of LaeA, VeA, and VelB in *A. ochraceus*, and also providing the vision on light regulating OTA biosynthesis mechanism.

Thus, we obtain the deletion mutants of *laeA*, *veA*, and *velB* of *A. ochraceus* and compare their characteristic for development, OTA biosynthesis and fungal virulence on pears. Deletion of *laeA* led to the dramatic reduction of conidiaspore, and deletion of laeA, VeA, and VelB led to the slowing down of growth rate. The biosynthesis of OTA was strongly regulated by LaeA, VeA, and VelB, for the production of OTA was decreased by three order of magnitude in the deletion mutants. All the three proteins affected the pathogenicity of *A. ochraceus* on pears. However, we could not confirm whether pathogenicity be related to OTA biosynthesis. Some studies were reported to prove the role of mycotoxin in fungal virulence (Barad et al., 2014), whereas others not (Ballester et al., 2015). It is meaningful to in-depth study the relationship among development, OTA biosynthesis and fungal virulence of *A. ochraceus* for exploring strategies of OTA contamination.

The mechanism of LaeA playing its regulatory role is unclear until now, although a number of studies referring to various fungi focus on LaeA. Being a member of velvet complex is only one of the mechanisms. The S-adenosyl methionine-binding site contained in LaeA presumably indicates its methyltransferase activity. Additionally, it has been suggested that this protein has been linked to changes in chromatin structure because loss of LaeA leads to increased hetero-chromatin marks and its often precise regulation of secondary metabolites (Bok and Keller, 2016). In this study, we focused on the regulatory role of LaeA on secondary metabolite biosynthetic genes for its widely accepted function. About 66.7% backbone genes in NRPS cluster were significantly regulated by LaeA, among which about 85.7% of the genes were down-regulated. In addition to backbone genes in PKS, Terperne, hybrid, and other clusters, 66.1% of the genes were significantly regulated, and 81.6% of differential expression genes were downregulated (Figure 7). These data proved the role of LaeA in secondary metabolite biosynthesis regulation, and deletion of laeA repressed the expression of many compounds as reported previously (Bok and Keller, 2004; Perrin et al., 2007). Although the structure of compounds corresponding to each cluster was not clear, this study would gain insights to the link between compounds and biosynthetic gene clusters.

In conclusion, results from this study have provided some evidence that velvet complex proteins (LaeA, VeA, and VelB) play important roles in morphology development, OTA biosynthesis and fungal virulence in *A. ochraceus*. And we further demonstrated LaeA widely affect gene expression of *A. ochraceus* genome, with a focus on secondary metabolites. The down regulation effect of LaeA was more than up regulation effect in secondary metabolism. Given the strong effect of *laeA*, *veA*, and *velB* on OTA biosynthesis, these genes could be designed as target sites to develop new strategies for OTA control and prevention.

## Data Availability Statement

All datasets generated for this study are included in the article/Supplementary Material.

## Author Contributions

YL and GW designed the experiment. GW, YW, FL, EL, JM, BY, and CZ performed the experiments. GW, LL, and HZ analyzed the data. GW wrote the manuscript.

### Conflict of Interest

The authors declare that the research was conducted in the absence of any commercial or financial relationships that could be construed as a potential conflict of interest.
