# Supplementary material for: Requirement of LaeA, VeA, and VelB on Asexual Development, Ochratoxin A Biosynthesis, and Fungal Virulence in Aspergillus ochraceus
Source: Front Microbiol. 2019 Nov 28;10:2759. doi: 10.3389/fmicb.2019.02759 (PMC6892948; doi:10.3389/fmicb.2019.02759)
Supplement: Supplementary file 1 [file Data_Sheet_1.docx]

**
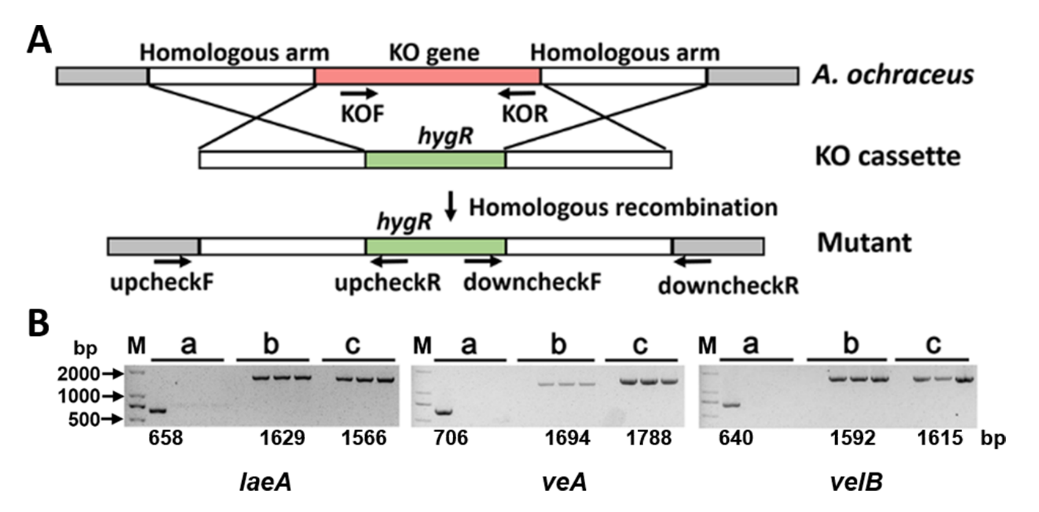
**

**Figure S1 Strategy and PCR identification of *laeA*, *veA* and *velB* gene deletion.** **(A)** Double homologous recombination strategy for deleting target gene by introducing the KO cassette. **(B)** PCR amplification verified validity of mutants. a, b and c represented fragments amplified by primer pairs KOF/KOR, upcheckF/upcheckR and downcheckF/downcheckR, respectively. Four lanes corresponded to each primer pairs were amplification from one WT and three transformant isolates.

**Table S1 Primers used for gene deletion cassette construction and transformant screening**

| **Primers** | **Oligonucleotide sequence (5’-3’)** | **Uses** |
| --- | --- | --- |
| hygR-F | GGAGGTCAACACATCAATGCCTATT | hygR amplification |
| hygR-R | CTACTCTATTCCTTTGCCCT |  |
| laeA-up-F | TGGAATGCCCATCTCAACCC | laeA deletion cassette |
| laeA-up-R | ACCAAAATAGGCATTGATGTGTTGACCTCCCGCCGTTTGGGAGTAGAAGT |  |
| laeA-down-F | CACTCGTCCGAGGGCAAAGGAATAGAGTAGCACATATACCAGGCGCGGAA |  |
| laeA-down-R | GTGACGCAGACGAGGAGATT |  |
| veA-up-F | ATCCTAGAAACCAACGGCGG | veA deletion cassette |
| veA-up-R | ACCAAAATAGGCATTGATGTGTTGACCTCCCGTTTGTCCGTCGAGTCCAT |  |
| veA-down-F | CACTCGTCCGAGGGCAAAGGAATAGAGTAGCCACAACCTATCCAAGCGGA |  |
| veA-down-R | GTACGGCCAAATGGTAAGCC |  |
| velB-up-F | GCACATCTCCCTTACGTGGT | velB deletion cassette |
| velB-up-R | ACCAAAATAGGCATTGATGTGTTGACCTCCGCCCGAGAACGGTACTAGATG |  |
| velB-down-F | CACTCGTCCGAGGGCAAAGGAATAGAGTAGGGGTGTCATTGAGAGCACGA |  |
| velB-down-R | GGTGAACAGGAGAAGCCGAA |  |
| LaeA-probe-F | AGGCTCTCGATGAGCTGATGCT | *laeA* probe for southern blotting |
| LaeA -probe-R | CGGAATGCAGTAGCACTGGCAT |  |
| veA-probe-F | ATGAAACCCTGGATGGTCGACT | *veA* probe for southern blotting |
| veA-probe-R | TAACGATCTTTGTAGAAACCAT |  |
| velB-probe-F | GTGAATTGTATCCGGGCCGAT | *velB* probe for southern blotting |
| velB-probe-R | TAACGATCTTTGTAGAAACCAT |  |

**Table S2 Primers used for RT-PCR amplilfication**

| **Primers** | **Oligonucleotide sequence (5’-3’)** | **Uses** |
| --- | --- | --- |
| GADPH-RT-F | TGCTCAAGTACGACAGCACC | RT-PCR amplification for the reference gene |
| GADPH-RT-R | CGAACTGGTCGTTGATGACC |  |
| AoFC_09697-RT-F | ACAGGGTCTATCGTCTCCGG | RT-PCR amplification for AoFC_09697 |
| AoFC_09697-RT-R | GCCATACTAATCTCCCCGGC |  |
| AoFC_09698-RT-F | CGCCACGTCAATAAGTCTCGG | RT-PCR amplification for otaA |
| AoFC_09698-RT-R | GTATGGAGCGTGCAGATCTG |  |
| AoFC_09699-RT-F | CTCGGCTACCTGCCTTCATG | RT-PCR amplification for otaB |
| AoFC_09699-RT-R | CAATGCCAACGCAATCAACG |  |
| AoFC_09700-RT-F | AGTCTTGCGCAAGCTACGAG | RT-PCR amplification for otaC |
| AoFC_09700-RT-R | CCGGACGTCATCCACTGTAG |  |
| AoFC_09701-RT-F | CCAGGACTCGTTCAGTCTCC | RT-PCR amplification for otaR1 |
| AoFC_09701-RT-R | GAGCACCCTGCGACATCATG |  |
| AoFC_09702-RT-F | CTCTCTACGGGGCGATTGTG | RT-PCR amplification for otaD |
| AoFC_09702-RT-R | CCTTGTACGCCTTAGCCAGG |  |
| AoFC_09703-RT-F | TATGGTCTCGCTACTCTGGCC | RT-PCR amplification for AoFC_09703 |
| AoFC_09703-RT-R | GTAGCAGTCGGATGCAGCAG |  |

**Table S3 Primer sequences used for qRT-PCR**

| **Primers** | **Oligonucleotide sequence (5’-3’)** |
| --- | --- |
| GADPH-qRT-F | TGCTCAAGTACGACAGCACC |
| GADPH-qRT-R | CTCGGCGAAGAACTGAACCT |
| AoFC_09697-qRT-F | CCGAATCGACTATGCCGATCC |
| AoFC_09697-qRT-R | GCTGGACGAATGGTCTTGAGG |
| AoFC_09698-qRT-F | GACCGAATCAGCTCACAGCAC |
| AoFC_09698-qRT-R | CGGACGAATGTCCTACTACCG |
| AoFC_09699-qRT-F | CCCAATCTGACCCCAGAACG |
| AoFC_09699-qRT-R | CTCGTCATTCAGGTATCCCCTG |
| AoFC_09700-qRT-F | CACCGCCGACATTATCACCTC |
| AoFC_09700-qRT-R | GCGATCATTCCGATCTCGACG |
| AoFC_09701-qRT-F | GGCTCAACAGCACAGTTCGAG |
| AoFC_09701-qRT-R | GTCATTACCAGCGCCGACAG |
| AoFC_09702-qRT-F | CTTCGAGGCGCTTCAGGATG |
| AoFC_09702-qRT-R | GCGCGCTCCTTCAATTGATTC |
| AoFC_09703-qRT-F | ACGCAATGAGCTACGGAACC |
| AoFC_09703-qRT-R | CTTGATGGCAGAATTGCGGG |
| AoFC_09543-qRT-F | GACAGCTTCTTTGCCATCGG |
| AoFC_09543-qRT-R | TCGAGCTGACGATCTTCCAG |
| AoFC_06119-qRT-F | CGAGCTGGATGACCTCTCAT |
| AoFC_06119-qRT-R | AGGATCCAATGGGACGAACG |
| AoFC_07015-qRT-F | CATTCTATATGCGACGGCTGG |
| AoFC_07015-qRT-R | CGCCTTCGTCGTATCGATATAC |
| AoFC_08050-qRT-F | GCCTTCCTTCCGTCTCCTATG |
| AoFC_08050-qRT-R | CATGGAGGATGTCGACAAGTG |
| AoFC_06120-qRT-F | GCTCTGTGGGCACTCATTCTC |
| AoFC_06120-qRT-R | GGCGGACCATGATATTTCCTCG |
| AoFC_09894-qRT-F | GTTGAGCCCTATGAGATCGCAG |
| AoFC_09894-qRT-R | CTCTTCTCTATGCTCGCATGG |
| AoFC_11716-qRT-F | GCTGCGATTACTTGCATGGG |
| AoFC_11716-qRT-R | ACGATAGGCTGACTGATCTCAG |
| AoFC_07623-qRT-F | CTGGTCTATCAACACAGCAAGC |
| AoFC_07623-qRT-R | GGCTGTAGTGTGCACGAGAC |
| AoFC_11426-qRT-F | CGCTTCACAGCGTCTATATCG |
| AoFC_11426-qRT-R | GAATCATCTCCGCGATGACG |
| AoFC_04810-qRT-F | GGCTTCAACACGTACGCTATC |
| AoFC_04810-qRT-R | GGTATGCGATTCGTTGGACG |
| AoFC_11183-qRT-F | CCTTCCTCCCTCTCGCATAC |
| AoFC_11183-qRT-R | CAGCTCTGCCAGAGACAGAAC |
| AoFC_05882-qRT-F | CCATGTTGACGCATCACAACC |
| AoFC_05882-qRT-R | CGACCCATGAGTAACGACGG |
| AoFC_03076-qRT-F | CTCTGTGGACTGTAGTTGCG |
| AoFC_03076-qRT-R | GTATCTTCGCAGATCTTCCGC |
| AoFC_08653-qRT-F | CCAGTGAGCCGATTCGAGAC |
| AoFC_08653-qRT-R | GGAATGGGGATAGTCTTGGGG |
| AoFC_11180-qRT-F | GCAGTTCATGAGCTACGTGG |
| AoFC_11180-qRT-R | GGTTGAGCTTCATCTCACGGTG |
| AoFC_04923-qRT-F | CTGCAGAACAATGCTCAGCG |
| AoFC_04923-qRT-R | CACATATTGTGCGTTGTATGCC |
| AoFC_05883-qRT-F | GCTAATCATGACACGACGTCTC |
| AoFC_05883-qRT-R | GTTGTCGTAAGTTGTCGAGGC |
| AoFC_06550-qRT-F | CGACTACTTGCCAGACTACATG |
| AoFC_06550-qRT-R | CTGGGTCTGATCTGGTGTACG |
| AoFC_07020-qRT-F | GGTGATCGAGCGACATAACG |
| AoFC_07020-qRT-R | CGATGTATCGTAGGGAGGCG |
| AoFC_07624-qRT-F | GAGATGTTGTGCACCGACGC |
| AoFC_07624-qRT-R | GTGACGTGACAAGCGAGAAG |
| AoFC_09529-qRT-F | CACAACTATTGGATGAGCTGCG |
| AoFC_09529-qRT-R | GTAGTCGCTTGCGATCGATC |
| AoFC_01940-qRT-F | GTGTTCTTCGTGCCATAGGC |
| AoFC_01940-qRT-R | TGCCCACGTATGTTGTGCAAG |
| AoFC_07483-qRT-F | GGTGACTGGTGAGGAGATCG |
| AoFC_07483-qRT-R | GATCTCAATCATCACGGACACG |
| AoFC_08067-qRT-F | GTATCAGTGGCGCTATGATTGC |
| AoFC_08067-qRT-R | GAAGATGCCATCATGCTGCAG |
| AoFC_01795-qRT-F | CGTCCTGACCCATAATGAGTG |
| AoFC_01795-qRT-R | GGATTGTAGGCGATGTCATGG |
| AoFC_05134-qRT-F | GATCCCATCCGACAGATTCGAC |
| AoFC_05134-qRT-R | CGTCTGCATCGCTTCTCTAGG |
| AoFC_07597-qRT-F | GCTTCATGCACAGTACCAGTTG |
| AoFC_07597-qRT-R | GAGAGGCATGCAGGTCTTGG |
| AoFC_04242-qRT-F | CCTCGTCTCCGATGAATGAACC |
| AoFC_04242-qRT-R | GCAATCTCCCCAGACGAGTG |
| AoFC_00328-qRT-F | GAGAAGCACGTCTATCCCAGC |
| AoFC_00328-qRT-R | CTCCGATCTCGCCAGATGAG |
| AoFC_10736-qRT-F | CCAGAGTGTCTAGTCTCAGCG |
| AoFC_10736-qRT-R | GAGTATGCTGCGAGGCAATC |
| AoFC_02280-qRT-F | GGAACAGTCAAGGACGGGTC |
| AoFC_02280-qRT-R | GCACGATACAGAGACGAGAGC |
| AoFC_01791-qRT-F | CGCTACTGGAAGAGGATCAATG |
| AoFC_01791-qRT-R | CCCATTCTCGTCAGTGAGCG |
| AoFC_02401-qRT-F | CAACTCTGCAGAGATCTCGCA |
| AoFC_02401-qRT-R | GCATAGGCAGCTGCAATCTC |
| AoFC_08990-qRT-F | GACGCCATTGATGCTGAGTAC |
| AoFC_08990-qRT-R | CGAACCTGTCCAACGCAATG |
| AoFC_06810-qRT-F | GAGGAGAACGGACGAGGATG |
| AoFC_06810-qRT-R | CATAAGCGACAGGTACTTCGC |
| AoFC_00237-qRT-F | GTCACATCGTCACGGTCTCTG |
| AoFC_00237-qRT-R | CAACGTGAGTCCTCCGATATCC |
| AoFC_06374-qRT-F | CGGAGAAGCATACCAGGAAGC |
| AoFC_06374-qRT-R | CTGTTAGCATCGCACGAACAG |
| AoFC_07038-qRT-F | CCGCATCTCTTGACGCCATC |
| AoFC_07038-qRT-R | GACAGACAGGTTAGATGGCTGG |
| AoFC_01818-qRT-F | CACTGGGACGGGTACTACTG |
| AoFC_01818-qRT-R | CAACGCTGTACAGGCGAGATG |
| AoFC_01462-qRT-F | CAGCGTTAGGCTGTCTCAGG |
| AoFC_01462-qRT-R | CATGCAGCGTCAATTGCGAC |
| AoFC_05416-qRT-F | CCTACGCAGCTGGTGTCTTG |
| AoFC_05416-qRT-R | CCAATCGCTGAGGGGATCTG |
| AoFC_09789-qRT-F | CCATCGCCAGAATCACATCCC |
| AoFC_09789-qRT-R | CAACTGTTCTGCACCCTCGATG |
| AoFC_06330-qRT-F | CTCTCTCCATCATGCGTGCTC |
| AoFC_06330-qRT-R | GATCGAAGACGTAGCTGGGTAG |
| AoFC_02377-qRT-F | CTTATGCAGCCAACGCCTTATC |
| AoFC_02377-qRT-R | CTATTCTCGCAGGCCAACAC |
| AoFC_02436-qRT-F | GAACCTGCAGTCCACAGTCC |
| AoFC_02436-qRT-R | GTCGTATTGGTCCTGCAAGG |
| AoFC_03569-qRT-F | GTACGATTCCACCGCATTGT |
| AoFC_03569-qRT-R | CTTCCTGCTCCACTCCGTGAC |
| AoFC_06064-qRT-F | GCACGGCTTGCAGGTTATTC |
| AoFC_06064-qRT-R | GGGGCGTCATCAGCATACTC |
| AoFC_07251-qRT-F | CATCAAGCAAGTGGCTACAGC |
| AoFC_07251-qRT-R | CGGTAGGGTAAGACGCTTCG |
| AoFC_07533-qRT-F | GCATCGGCGCTCTTGTCATTC |
| AoFC_07533-qRT-R | CGCGAATTAACTGCTCCTGC |
| AoFC_08701-qRT-F | GCACATCGGCTGATATCGAG |
| AoFC_08701-qRT-R | GAACCCGACCAACTGTCCAATG |
| AoFC_10219-qRT-F | GCGAGAATCCTCAAGCACAG |
| AoFC_10219-qRT-R | CAGAGTTCACTGCGTGAAGG |
| AoFC_10777-qRT-F | CTGACGGTGTCGACTCTCTG |
| AoFC_10777-qRT-R | GAATGTCCTCGCGAACTTGAG |
| AoFC_06430-qRT-F | CAGCCCTCTACTTCGAGTGTC |
| AoFC_06430-qRT-R | GAGGTCGGCGTAGACTGTTG |
| AoFC_05376-qRT-F | GACTGTCTTGACTTCGTCTGC |
| AoFC_05376-qRT-R | GCTCGTCCCGTTACCAACTG |
| AoFC_05377-qRT-F | GTCGAAGCTCATCGAGCAGC |
| AoFC_05377-qRT-R | CTCGATCTTGAGACGACGAGC |
| AoFC_02449-qRT-F | GTCTACGACGCGAGAATGACAC |
| AoFC_02449-qRT-R | GAGATGGTCACACTATCCGGG |
| AoFC_03542-qRT-F | GGGAGCACACTCAAGCTCATTG |
| AoFC_03542-qRT-R | CTTGACTCTGGAACACATGCTC |
| AoFC_00340-qRT-F | GTTCACCATCGAGATGTCGTG |
| AoFC_00340-qRT-R | CGGAAGTCGTGTGTTCATGC |
| AoFC_10399-qRT-F | CGACTCCATCCTCGCCATTC |
| AoFC_10399-qRT-R | CGAAGGGTGACTCTTCGGAC |
| AoFC_07675-qRT-F | GGTGGACCTGACATCACTTGG |
| AoFC_07675-qRT-R | CCCAGCTGCCATCTTCTTCC |
| AoFC_03000-qRT-F | GGTTGCACGGCAGAGTATGC |
| AoFC_03000-qRT-R | GCCAGCTGCTCCATCTCAATG |
| AoFC_02451-qRT-F | CGGAGAGAATGTCAGTCGTGG |
| AoFC_02451-qRT-R | CAGTTCTCAGCATTAGGCGAGG |
| AoFC_02999-qRT-F | GGATGCTGTGGATGTCGAGTC |
| AoFC_02999-qRT-R | CTGTCGGAAGGTGGCTCTAG |
| AoFC_07667-qRT-F | GAGCAGCCCTAATCCGAGAC |
| AoFC_07667-qRT-R | CAGGCTATAGCTGCTGCAGTAC |
| AoFC_00343-qRT-F | GGCTGGAGTAACTGGTATGTCG |
| AoFC_00343-qRT-R | CAATCCTCCAGTACCATGTCCC |
| AoFC_00016-qRT-F | CGTTCAGGTCTTACTGGTTCGC |
| AoFC_00016-qRT-R | CCCCAAACCCTCGATAATATGC |
| AoFC_00120-qRT-F | CGAATGTTGGGTGAATCACTGC |
| AoFC_00120-qRT-R | GATTCAAGTGTGCGATTCCACC |
| AoFC_00127-qRT-F | CCTTGACCAGGTTCATGCATG |
| AoFC_00127-qRT-R | CATCGCCCTTATCAACCTGTTC |
| AoFC_03008-qRT-F | GCTCCTACGAATGTGTTGCCC |
| AoFC_03008-qRT-R | CGTTCGGAATTGTGGGATGC |
| AoFC_05313-qRT-F | GCAGAGCAGGGAGAGGACTC |
| AoFC_05313-qRT-R | GAGAGAATGCCCACCCAAGTG |
| AoFC_09572-qRT-F | CGCCATGAACAGTCCGTCTAG |
| AoFC_09572-qRT-R | GGGGATACATGTGGTGACTGTG |
| AoFC_09699-qRT-F | CCCAATCTGACCCCAGAACG |
| AoFC_09699-qRT-R | CTCGTCATTCAGGTATCCCCTG |
| AoFC_01587-qRT-F | CGATCGGCTATGCATTGCAAC |
| AoFC_01587-qRT-R | GGAGAGCGATGCATGGATGAG |
| AoFC_04140-qRT-F | GCCAGTCTACCTTCCTGATCCG |
| AoFC_04140-qRT-R | GTTGGTTCTCAGGATCGGTGG |
| AoFC_05451-qRT-F | CGCTTCGACAATGTATCGCTTG |
| AoFC_05451-qRT-R | GCCTTGAAGAGCTTCGATTCCC |
| AoFC_10494-qRT-F | GATCGGATACAGCTTCTGGCG |
| AoFC_10494-qRT-R | GTATCTCAGTCGCTCGCCTCTG |
| AoFC_01758-qRT-F | GATGGTCGTTGGGGGTATGG |
| AoFC_01758-qRT-R | CGATCTTGAGCGTGCAATCC |
| AoFC_04183-qRT-F | CGCCAGCCTCGAATATCTCTG |
| AoFC_04183-qRT-R | GTCAAGATGTTGCTGGTCCACC |
| AoFC_07656-qRT-F | CGACCTGTACTGCTACTACGTG |
| AoFC_07656-qRT-R | GAACAGACCCATCGACTTGTGC |
| AoFC_05939-qRT-F | GATTGATCTGCGGTGGTTCG |
| AoFC_05939-qRT-R | GAAGTCGATCGATAGGCTCTCG |
| AoFC_06354-qRT-F | CTCACACGGGAAGCTCTGAAG |
| AoFC_06354-qRT-R | CGTCAGACGGAGATGGATGC |
| AoFC_09594-qRT-F | CCACCGACTGACTCTATCAGAC |
| AoFC_09594-qRT-R | CTGCCGAGAATATCAACTCCG |
| AoFC_02208-qRT-F | CCTGACCCTAGCACAATTGATG |
| AoFC_02208-qRT-R | CAGGCTCAAGGCAACTCTTC |
| AoFC_02222-qRT-F | GCCTGTTCAACGAGAGATCCC |
| AoFC_02222-qRT-R | CACCCAATTCCCCCAGTCAC |
| AoFC_04524-qRT-F | GCACCAGTGAAGACGATCCTC |
| AoFC_04524-qRT-R | GCAGTCCCATTCCATGACACC |
| AoFC_06088-qRT-F | CCCTCTCCTTGTTGCATACAC |
| AoFC_06088-qRT-R | GTACACCCATAGAGAACCCCCC |
| AoFC_07152-qRT-F | CATGAACGGGGACTCTCTTCAG |
| AoFC_07152-qRT-R | GAGACCATTGACCGTCTCGATG |
| AoFC_07333-qRT-F | GCGTATCTTCGCCAGATCTACG |
| AoFC_07333-qRT-R | GCATGAGGCACAGGTGGATC |
| AoFC_09475-qRT-F | CATGTCGCCTGCTGACTCTG |
| AoFC_09475-qRT-R | GAGATGGCGATGAGTCGCTC |
| AoFC_10722-qRT-F | GTCCTTCCGAGACTGCGAATC |
| AoFC_10722-qRT-R | CTACTCAACCCGTCCCGTCTC |
| AoFC_02581-qRT-F | CTAGATGATCGCGGTTGGAAC |
| AoFC_02581-qRT-R | GTCGTCTGTATTGGAAGCGCTC |
| AoFC_02511-qRT-F | CATAGCTCTGGGACAACAGGC |
| AoFC_02511-qRT-R | GTTGGGACGAGGAGAAGCAG |
| AoFC_02427-qRT-F | GCAATCCACACCTCTGCCAC |
| AoFC_02427-qRT-R | GTGCGAGAAGTTCACGGATGC |
| AoFC_00265-qRT-F | GTTGCATCACAGCCTGATGC |
| AoFC_00265-qRT-R | CCAGCCGTAACGCCATCTTC |
| AoFC_06267-qRT-F | CCACACACGTCCATCACTCTG |
| AoFC_06267-qRT-R | CACGACTGGGGACGATCAAC |
| AoFC_06282-qRT-F | CCCATCAGTGATAGCCTCTACG |
| AoFC_06282-qRT-R | CCGTGTGACATACTTCCATCGC |
| AoFC_10892-qRT-F | GACGGCTCTTATGCGACGAAG |
| AoFC_10892-qRT-R | CTCGACCTTCCAGATCCAGAG |
| AoFC_00078-qRT-F | GTGCCATCGTTACTCTGCCG |
| AoFC_00078-qRT-R | CAGCAATGCCGTGGTGAGTAG |
| AoFC_06279-qRT-F | GGGTCTGGGTTGCACATATCTG |
| AoFC_06279-qRT-R | CCCTTCACCAACAGCTCTCC |
| AoFC_11745-qRT-F | CAGCGTCGTTACGGAATTGG |
| AoFC_11745-qRT-R | CACATTCGCTTGAGCCTAGCAG |
| AoFC_02497-qRT-F | CGGGTGCATAGCTCGGATATAC |
| AoFC_02497-qRT-R | GCTTGGTCCCCAGTATTGAACC |
| AoFC_10426-qRT-F | CTCGTATTCTCTACCCTGCACC |
| AoFC_10426-qRT-R | GGACTTGGTGATCGAGTTGGAG |
| AoFC_11068-qRT-F | CTTCAGGCTGCTACATTGATCG |
| AoFC_11068-qRT-R | CCCAAGTCTCTGCCTCATCG |
| AoFC_08037-qRT-F | CATCTGTCTCAACCGAAGCG |
| AoFC_08037-qRT-R | GCTTGGAGAGGTCTGCTTGTAG |
| AoFC_03784-qRT-F | CCAACACGCGACTCCTCAATTG |
| AoFC_03784-qRT-R | GCCGCTCTCGTCGAGAATATG |
| AoFC_08580-qRT-F | GGGTAATCTTCGTCGCAGTGC |
| AoFC_08580-qRT-R | GCGCAAGGACTCTTGAGAAG |
| AoFC_06991-qRT-F | CGTCGAAGGAGAACGCATGG |
| AoFC_06991-qRT-R | CCACACCTTCGATGACCAGC |
| AoFC_02918-qRT-F | GCTCACTTGAGCTCCAGCAC |
| AoFC_02918-qRT-R | GGATGTGTCGTAGGTCATGAGG |
| AoFC_02419-qRT-F | GCGAGAGCTTCTCGGATGTTC |
| AoFC_02419-qRT-R | GATCCGACATCTCCTGTAGCG |
| AoFC_04637-qRT-F | CAGCGTGATATTCCATGCCG |
| AoFC_04637-qRT-R | GTAGTGCAATGCCTTCCGCC |
| AoFC_07882-qRT-F | CGGTACGAGCAAGATGGGAG |
| AoFC_07882-qRT-R | GTCCGTGGTCAAGACACGAG |
| AoFC_08034-qRT-F | GCCGATCACTTCAGATTCGCTG |
| AoFC_08034-qRT-F | CCCGTCACGACTTGACATCG |
| AoFC_08406-qRT-F | GGGTTCTTGGGTGCTCACATC |
| AoFC_08406-qRT-R | CCTCGTCCCAGAATCCGTAG |
| AoFC_08538-qRT-F | GACTCCATCCGCGCTATCAG |
| AoFC_08538-qRT-R | GTATGTAGCTGGGAGCGTGC |
